# Percutaneous endoscopic lumbar discectomy: minimally invasive technique for multiple episodes of lumbar disc herniation

**DOI:** 10.1186/s12891-017-1697-8

**Published:** 2017-08-01

**Authors:** Kyung-Chul Choi, Jin-Sung Kim, Dong Chan Lee, Choon-Keun Park

**Affiliations:** 1 Department of Neurosurgery, the Leon Wiltse Memorial Hospital, Anyang, South Korea; 2 Department of Neurosurgery, Seoul St Mary’s Hospital, College of Medicine, The Catholic University of Korea, 222 Banpodaero Secho-gu, Seoul, 137-040 South Korea; 3 Department of Neurosurgery, the Leon Wiltse Memorial Hospital, Suwon, South Korea

**Keywords:** Adjacent segment degeneration, Open lumbar discectomy, Percutaneous endoscopic lumbar discectomy, Transforaminal

## Abstract

**Backgrounds:**

Although open lumbar discectomy is a gold standard surgical technique for lumbar disc herniation (LDH), surgery-induced tissue injury may actually become a source of postsurgical pain. Percutaneous endoscopic lumbar discectomy (PELD) is introduced as a minimal invasive spinal technique for LDH. The PELD has gained popularity and shown successful results. The authors report the clinical usefulness of the PELD technique in two patients with the serial multilevel LDHs.

**Case presentations:**

A 32-year-old man suffered from radicular pain at the L5 dermatome due to the down migrated soft LDH at the L4–5 level. The PELD was performed to remove the ruptured fragments, yielding a complete decompression of the L5 nerve root. Four years later, he visited the clinic because of right leg radiating pain along the S1 dermatome. An MRI scan revealed the LDH at the L5-S1 level. The PELD with foraminoplasty was also performed successfully at the L5-S1 level. Two months after the second PELD, he visited the clinic again because of severe pain along the left L4 dermatome; consequently, the PELD was also performed at the L3–4 level without any complications. A 34-year-old man presented with radiating pain in the back and both legs at the L5 dermatome. The MR images show a disc extrusion at the L4–5. The patient underwent the PELD at the L4–5 via the left approach. After the PELD, the back and leg pain both improved. One year later, the patient suffered from severe pain in the back and the left anterior thigh. The MR images show a left paramedian LDH at the L2–3. After the PELD was performed at the L2–3, the pain was relieved. The final MR images show no signs of any aggravated degeneration of the intervertebral discs or the facet joints at all of the treated levels.

**Conclusion:**

When multiple episodes of LDH occur in a patient’s life span, PELD could be considered as an alternative good technique to treat LDH in each step by preserving normal anatomic structures.

## Background

Although the open lumbar discectomy (OLD) is a gold standard surgical technique for the lumbar disc herniation (LDH), iatrogenic damage on the paraspinal muscles, ligaments and facet joints, with reduced disc height, segmental instability and retrolisthesis, may become a source of postsurgical pain [1–3]. Percutaneous endoscopic lumbar discectomy (PELD) can be performed under local anesthesia. It has many advantages such as less paraspinal muscle trauma, preserving facet joint, and with smaller surgical wound while minimizing postoperative instability [4, 5]. There are few reports about the requirement of discectomy at adjacent segment degeneration (ASD) after lumbar discectomy. Repetitive OLD for ASD may affect postoperative instability, back pain, and surgical satisfaction. The experiences of sequential PELD procedures for the multilevel LDHs in two patients is reported.

## Case presentation

### Case 1

**Fig. 1:**
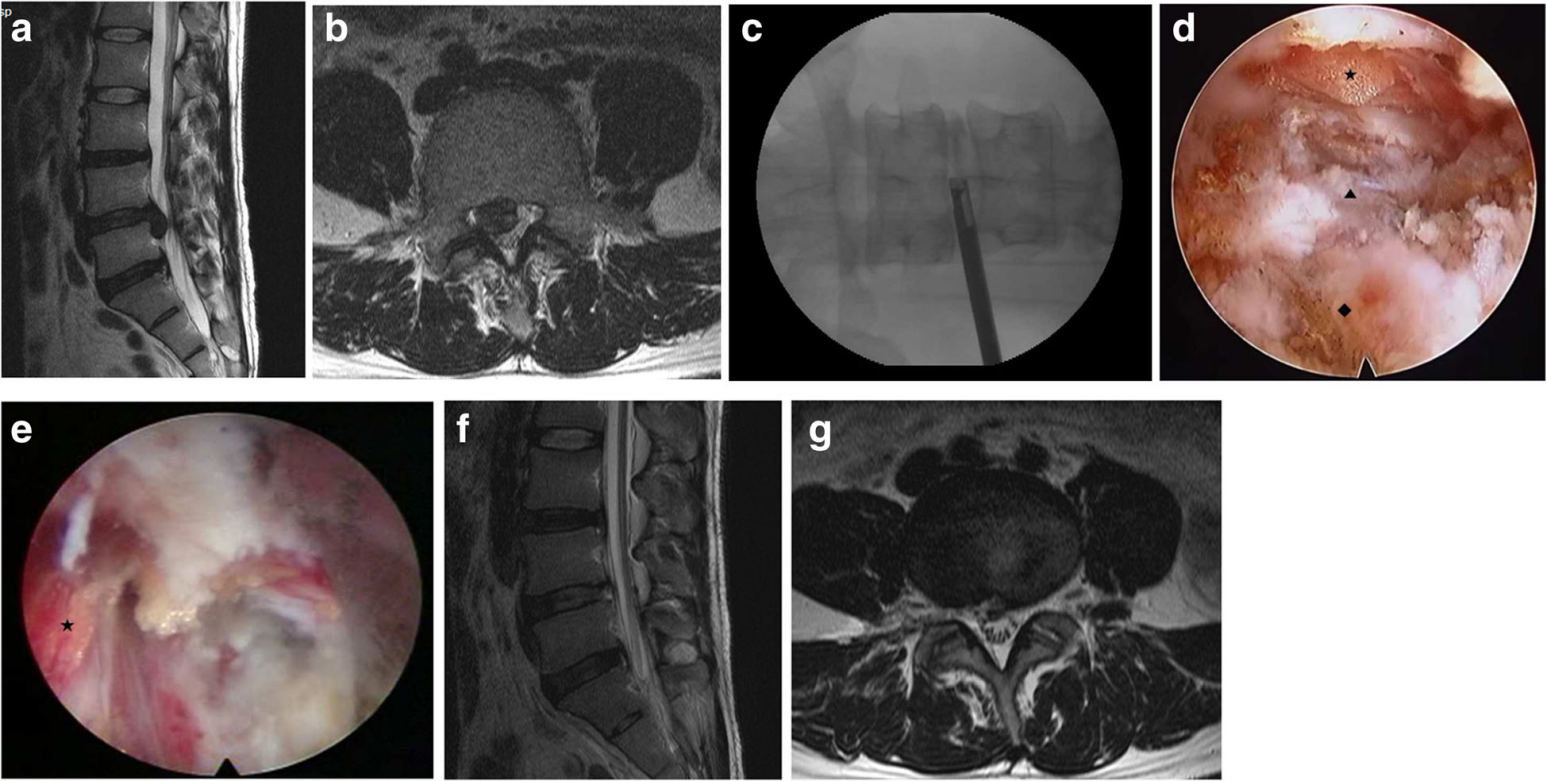
T2-weighted parasagittal magnetic resonance (MR) images showing down-migrated disc herniation at the L4–5 (**a** and **b**); after first PELD (**c**). After removing the nucleus pulposus (◆) and cutting the annulus (▲), the epidural space (★) is found to decompress (**d**). Pulling out the working cannula, the exiting nerve root (★) is found (**e**). MR images demonstrating complete removal of herniated disc (**f** and **g**)

**Fig. 2:**
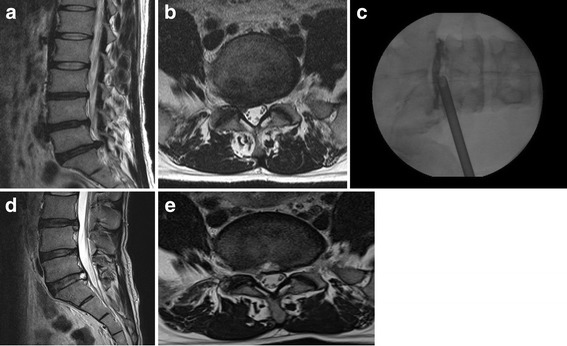
Four years later, MR images showing disc extrusion at the L5-S1 (**a** and **b**). After second PELD (**c**), and MR images showing complete decompression (**d** and **e**)

**Fig. 3:**
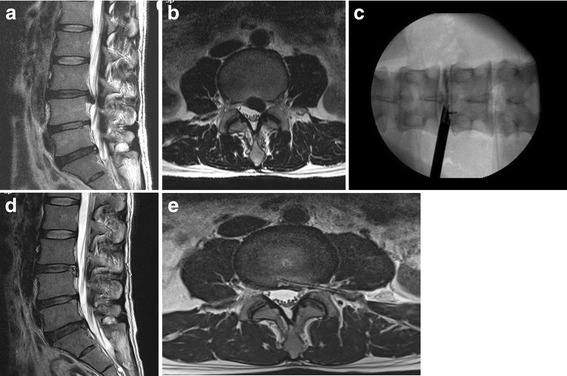
Two months later after second PELD: MR images showing down migrated disc herniation at the L3–4 (**a** and **b**). After third PELD (**c**), MR images showing complete removal of herniated disc and catheter of external drainage in the epidural space (**d** and **e**)

A 32-year-old man suffered from right gluteal, thigh and calf pain along the L5 dermatome for two months. The manual muscle test for the right great-toe dorsiflexion and the ankle dorsiflexion showed grades III and IV, respectively. The magnetic resonance (MR) images demonstrated the disc extrusion and the down migrated disc herniation at the L4–5 level (Fig. 1a and b). Although he underwent a steroid epidural injection and consumed medications, the pain did not improve. The PELD procedure was performed in the prone position under local anesthesia, whereby the patients communicated with the surgeon during the entire procedure. The skin entry point was determined as 13 cm from the midline. After the infiltration of the entry point with local anesthetics, an 18-gauge spinal needle was introduced under flouroscopic guidance. The needle tip was positioned at one point of the medial pedicle line on the anteroposterior fluoroscopic projection and at the posterior vertebra line on the lateral projection. Next, an epidurogram was performed using contrast media to confirm the locations of the exiting root and the traversing root. After the spinal needle was inserted into the disc, the nucleus pulposus was stained blue with a 1 ml mixture of contrast media and indigocarmine for the discography. A guide wire was inserted through the spinal needle, and a cannulated obturator was inserted along the guide wire. A bevel ended working cannula was inserted into the disc along the obturator, followed by the removal of the obturator (Fig. 1c). The pathologic nucleus was stained for easy discrimination under the endoscopic view. The blue stained disc was removed using endoscopic forceps. An observation showed that the inflamed nucleus was anchored by the annular fissure. The herniated disc and the fibrotic scar tissues were released and removed using endoscopic forceps and a radiofrequency. If necessary, annulus and posterior longitudinal ligament were resected for removal of herniated disc fragment (Fig. 1d). Pulling out the working cannula, the exiting nerve root was found (Fig. 1e). After the PELD, the visual analogue scale (VAS) scores of the back and leg pain improved from 6 and 8, respectively, to 2 and 1, respectively. Postoperative MR images (Fig. 1f and g) show the complete removal of the ruptured disc fragment. The patient was discharged on the day after the PELD; three days later, he returned to work. His improved symptoms had been maintained. Four years later, he visited the clinic because of right-leg radiating pain along the S1 dermatome. MR images revealed soft disc herniation at the L5-S1 level (Fig. 2a and b). Although he underwent an S1 nerve-root block, the pain was sustained. The PELD with foraminoplasty (Fig. 2c) was also performed successfully at the L5-S1 level (Fig. 2d and e). Two months after the second PELD, the patient visited the clinic again because of severe pain along the left L4 dermatome. On the left side, the straight leg raising test was positive at 30 degrees, and the dorsiflexion of the left ankle was reduced to grade IV of the manual muscle test; additionally, hypesthesia in the left L4 dermatome was noted. MR images showed the disc extrusion and the down migration at the L3–4 (Fig. 3a and b). The symptom sustained, although he underwent epidural blocks twice and took pain control medications for 6 weeks. The PELD was also performed at the L3–4 level without complications (Fig. 3c). After the removal of the herniated disc, a drain tube was inserted for the control of the epidural bleeding. MR images showed a complete removal of the herniated disc (Fig. 3d and e). Four days after the PELD, the patient commenced work. Three years after the third PELD, the patient worked independently, and his VAS scores for back and leg pain are 2 and 1, respectively.

### Case 2

**Fig. 4:**
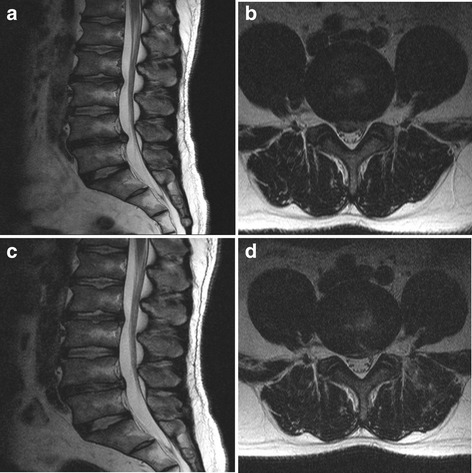
MR images showing broad based central disc extrusion at the L4–5 (**a** and **b**). After the PELD, MR images showing sound decompression (**c** and **d**)

**Fig. 5:**
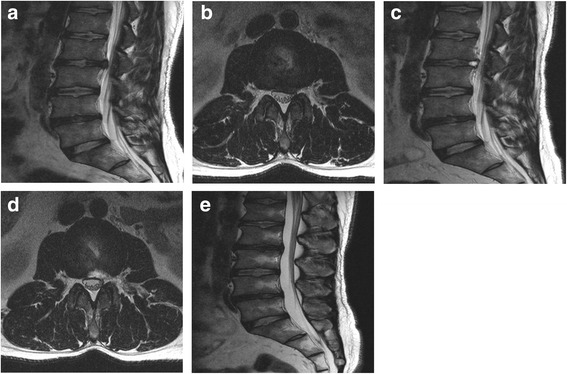
One year later, MR images showing left paramedian disc herniation at the L2–3 (**a** and **b**). After the PELD, MR images showing complete removal of the disc (**c** and **d**). Eighteen months later after second PELD, MR image (**e**) showing no significant changes of the disc height or any disc degeneration aggravation in comparison with initial MR images

A 34-year-old man presented with back and both leg radiating pain as an L5 dermatome. MR images showed a disc extrusion at the L4–5 (Fig. 4a and b). The patient underwent the transforaminal PELD at the L4–5 for which the left approach was used. After the PELD, the VAS scores of the back and leg pain improved from 7 and 7, respectively, to 3 and 2, respectively (Fig. 4c and d). One year later, the patient suffered from severe back and left anterior thigh pain. Although he underwent three epidural steroid injections, the pain was sustained. MR images showed a left paramedian disc herniation at the L2–3 (Fig. 5a and b). After the PELD at the L2–3, the pain was relieved. MR images showed a complete removal of the herniated disc (Fig. 5c and d). Eighteen months after the second PELD, the follow-up MR images showed no significant changes of the disc height or any degeneration (Fig. 5e).

## Discussion

Open lumbar discectomy (OLD) is still regarded as a standard technique for refractory lumbar disc herniation. The incidence of postoperative mechanical back pain following the OLD is not uncommon. Parker et al. [6] reported that 32% of patients suffered above-moderate back pain after the OLD, and 9% suffered severe back pain and subsequently underwent fusion surgery. A recent study on the long-term outcomes of the OLD shows that the outcome deteriorates over time. Worsening of clinical outcome was correlated with radiologic degeneration at operated segment [7]. The discectomy causes a narrowing of the disc space, leading to an overloading of the facet joints. Chronic facet joint pain originates from the extended removal of a disc and the consecutive reduction of the disc height, ant it potentially leads to the progressive disruption of spinal instability [8]. The iatrogenic muscle injury is associated with persistent back pain [9, 10]. Kawaguchi et al. [11, 12] noted that muscle degeneration occurred immediately after surgery.

Postoperative epidural adhesion and scar formation commonly develop after the OLD. Many surgeons have tried to preserve more ligamentum flavum to increase the clinical outcome of the lumbar discectomy [13, 14]. Preserving the ligamentum flavum decreases the rates of epidural fibrosis and perioperative complication. In PELD, an epidural scar or adhesion is not detected by MRI and operative field [15].

Since Kambin introduced the contemporary endoscopic discectomy technique [16], the technique and instruments of the PELD have markedly evolved [17]. Surgical outcomes of PELD have become comparable to those of open-discectomy technique [4, 5], including less paraspinal muscle trauma, no bone removal, and little epidural bleeding. In addition, disc height and foraminal height are less decreased in PELD than those in OLD [18].

The PELD is advantageous because it avoids the need for the nerve-root retraction as well as its preservation of the lamina, facet joint, and posterior ligament structures. An excessive retraction or manipulation of neural structures in a narrow space can cause paresis.

The rate of reoperation for adjacent segment disease after OLD was 4% in a study of 751 patients [19]. The incidence of adjacent level discectomy was 1.9%. Time to reoperation for ASD occurred over a mean of 3.11 years. It has been suggested that the annual reoperation rate for ASD is 1.35% [19]. Rostral ASD requires more common caudal ASD. It cannot exactly clear explain the cause of adjacent segment disc herniation. It might be due to natural course/aging process. According to biomechanical study, intradiscal pressure and intersegmental rotation of rostral segment are increased after discectomy. Rostral segment increases the anteroposterior translation in flexion and lateral translation in left lateral bending [20]. In the 5-year follow-up results, 12.4% of the patient underwent reoperation at operated level or other lumbar level after PELD. The rate of reoperation is similar to that (13.7%) after OLD [21]. PELD for recurrent disc herniation has yielded favorable outcomes [22, 23]. By preserving paraspinal muscle and avoiding iatrogenic facet injury, PELD is superior to conventional OLD with shorter hospital stay, lesser postoperative pain, and less intraoperative blood loss. Hur et al. [24] have reported single-portal dual PELDs for two-level concurrent symptomatic disc herniation. By using only one skin entry point, the ability to adjust the trajectory angle makes it possible to remove disc herniations at different levels. They have suggested that the application of single-portal dual PELDs is appropriate for unilateral radicular pain, same-side disc herniation, and downward migration of lower lumbar disc.

The PELD learning curve is usually perceived to be steep. But, Lee and Lee [25] reported learning curve is acceptable with relatively low failure and complication rates of 7.8% and 3.9%. Choi et al. reported the result of large PELD cases. Revision rate is 4.3% for incomplete removal of herniated disc, recurrence and remnant pain [26].

Although the PELD can allow a patient to return to work early and provide high-satisfaction surgical results, its application is limited in soft disc herniation without spinal stenosis. Also, the appropriate disc height and an intervertebral foraminal dimension should be secured.

## Conclusion

The PELD that avoids the occurrence of the iatrogenic normal-tissue injury may be an ideal surgical technique for the LDH. Based on the preservation of the normal anatomic structure, its usefulness could be maximized regarding the serial multilevel LDHs of a patient.

## Abbreviations

ASD: Adjacent segment disease
LDH: Lumbar disc herniation
MR: Magnetic resonance
OLD: Open lumbar discectomy
PELD: Percutaneous endoscopic lumbar discectomy
VAS: Visual analogue scale
